# A PSO-Powell Hybrid Method to Extract Fiber Orientations from ODF

**DOI:** 10.1155/2018/7680164

**Published:** 2018-01-21

**Authors:** Zhanxiong Wu, Xiaohui Yu, Yang Liu, Ming Hong

**Affiliations:** ^1^School of Electronic Information, Hangzhou Dianzi University, Hangzhou, China; ^2^Department of Systems Medicine & Bioengineering, Houston Methodist Hospital, Houston, TX, USA; ^3^Department of Biomedical Engineering, University of Houston, Houston, TX, USA

## Abstract

High angular resolution diffusion imaging (HARDI) has opened up new perspectives for the delineation of crossing and branching fiber pathways by orientation distribution function (ODF). The fiber orientations contained in an imaging voxel are the key factor in tractography. To extract real fiber orientations from ODF, a hybrid method is proposed for computing the principal directions of ODF by combining the variation of Particle Swarm Optimization (PSO) algorithm with the modified Powell algorithm. This method is comprised of the global searching ability of PSO and the powerful local optimizing of Powell search. This combination can guarantee finding all the diffusion directions without applying sliding windows and improve the accuracy and efficiency. The proposed approach was evaluated on simulated crossing-fiber datasets, Tractometer, and in vivo datasets. The results show that this method could correctly identify fiber directions under a range of noise levels. This method was compared with the state-of-the-art methods, such as modified Powell, ball-stick model, and diffusion decomposition, showing that it outperformed them. As to the multimodal voxels where different fiber populations exist, the proposed approach allows us to improve the estimation accuracy of fiber orientations from ODF. It can play a significant role in the nerve fiber tracking.

## 1. Introduction

At present, tractography based on diffusion-weighted magnetic resonance imaging (DWI) is the only noninvasive tool to obtain information on the neural architecture of the human brain white matter (WM) in vivo. The structural connectivity inferred from tractography is critical for understanding the functional coupling between cortical regions of the brain and for the characterization of neurodegenerative diseases and for medical applications [1–4]. In deterministic tractography, it is the important step to resolve the fiber orientations populated in each imaging voxel.

In current literature, there are three mathematical models applied to retrieve fiber orientations from DWI raw datasets: apparent diffusion coefficient (ADC), diffusion tensor (DT), and ODF. However, the local maxima of ADC profile do not necessarily coincide with the underlying fiber directions, making the extraction of orientation information difficult [5–7]. This is due to the nature of the ADC measurement which is the projection of spin displacements onto the diffusing gradient axis. The limitation of the DT model is the Gaussian diffusion assumption, which implies that there can only be a single-fiber population per voxel [8–10]. It is known that many voxels have low diffusion anisotropy due to the crossing, branching, and fanning of multiple fibers [11–14]. The ODF is defined as the radial projection of the spherical diffusion function, which is a function on the unit sphere describing the probability averaged over the voxel that a particle will diffuse into any solid angle [15, 16]. As a spherical function, ODF has its local maxima aligned with the underlying fiber directions at every voxel. Until now, ODF is most widely employed to determine the fiber orientations with high angular resolution.

As the water molecules in WM tissues tend to diffuse along fibers when contained in fiber bundles, the principal directions of ODF agree with the true synthetic fiber directions [17]. The ODF field is promising for the estimation of neuronal fibers. The orientation of a particular fiber population could be estimated by finding the peaks of the corresponding reconstructed ODF. For this reason, a major focus within the DWI community has been directed at developing methods to compute the ODF. Although diffusion spectrum imaging (DSI) was firstly developed to image complex distributions of intravoxel fiber orientations with a more detailed, complete, and accurate view of WM architecture locally, the time-consuming MRI signal sampling restricts its application [18]. Currently, Q-ball imaging (QBI), constant solid angle QBI (CSA-QBI), and diffusion orientation transform (DOT) are constantly used to construct the ODF because they are more time-saving than DSI [15, 16, 19]. However, there is still an important issue to be solved in the neural fiber tracking from ODF. That is how to extract the fiber directions from ODF accurately with high angular resolution. In general, there are two kinds of methods to extract fiber orientations from ODF, and the first is to resolve the ODF to multicompartment partial volume models, and the other is to directly search the peaks of ODF.

The method of diffusion decomposition obtains fiber orientations by decomposing ODF into standard component ODFs [20]. The advantage of the approach is that a fiber vector can be easily represented by the direction of component ODF but not by spherical harmonics. The decomposition algorithm provides a sparse solution to improve the ability in resolving crossing fibers and to avoid false fibers as encountered in diffusion deconvolution. Ball and sticks model is a model-fitting approach, which decomposes HARDI signals into isotropic and anisotropic diffusion components directly [21]. However, the two models suffer from the shortcomings regarding model selection. We must determine the number of diffusion compartments with a priori structural knowledge of each voxel and must use nonlinear optimization to obtain the fiber orientations. What is more, the two methods are sensitive to noise and to the number of HARDI measurements. Essentially, the two models are ill-posed inverse problems.

The ODF, as a probability distribution function, should be nonnegative. As the principal directions of ODF are consistent with fiber orientations, extracting the fiber orientations from ODF could be boiled down to a multimodal optimization problem. At present, several methods exist to extract ODF's maxima, such as finite difference method, Powell's method, and spherical Newton's method. By searching for local maxima of persistent angular structure (PAS) using Powell's method, the orientations of WM fibers were revealed [22]. Tournier et al. estimated the orientation of a particular fiber population by finding the peak of the corresponding reconstructed ODF using a spherical Newton's method [23]. This method has the merit of high convergence speed, but it is susceptible to the position of the starting point. In the iteration not only gradient vectors and their modulus but also Hessian matrix and its inverse matrix are needed to be computed over and over again. This is quite time-consuming and memory-consuming. Sequential quadratic programming (SQP) is an iterative method for nonlinear optimization. It usually is used on the mathematical problems for which the objective function and the constraints are twice continuously differentiable [24]. But SQP was found to introduce biases in the peak distributions via the constraints.

In order to find all the fiber-along vectors from ODFs and improve the precision of multipeak searching, in this work, we have introduced a novel methodology to estimate the fiber directions directly from ODF based on PSO-Powell hybrid algorithm. In this method, the global search ability of PSO is combined with the strong local search ability of modified Powell algorithm. This combination can not only improve the solution accuracy but also speed the searching at the same time. Only using the function value information without the need to calculate derivatives makes it very useful to solve ODF optimization. It can correctly retrieve the orientations corresponding to underlying intravoxel fibers populations. Results on the simulated datasets, Tractometer, and in vivo HARDI datasets illustrate the effectiveness of the proposed approach.

## 2. Methods

### 2.1. ODF Construction

In the past decades, respectable researchers have tried to extract fiber orientations with high angular resolution from raw DWI datasets. However, image acquisition under clinical conditions with limited measurement time faces the problem of poor spatial and angular resolution and the technique's high susceptibility to noise [25–27]. The advent of HARDI has provided the chance to delineate multifiber pathways effectively and efficiently by ODF. Essentially, ODF is a function of two angular variables *θ* and *φ* as (1). The equation expresses diffusion probability in the direction (*θ*, *φ*) of water molecules contained in WM tissue:

(1) $$\begin{array}{c} dODF\left(\;\theta ,\varphi \;\right)=\overset{\infty }{\underset{0}{\int }}P\left(\;r\left(\;\theta ,\varphi \;\right)\;\right)r^{2}dr, \end{array}$$

where *r* is the displacement radius, *θ* is the azimuth angle, and *φ* is the elevation angle in spherical coordinate. In this work, we applied the PSO-Powell hybrid method to the ODF fields which were constructed with QBI, CSA-QBI, and DOT. In order to be able to delineate fiber crossings even at low angles and avoid unnecessary loss of angular resolution, we choose a high SH order of 8 for ODF constructions in CSA-QBI and DOT. Higher-order spherical harmonics are necessary to resolve fibers that are separated by small angles but also introduce noise [28–30]. We applied a set of 724 directions evenly distributed on a unit sphere to evaluate the ODFs of the testing HARDI datasets.

After ODF fields have been constructed, it is crucial to resolve the principal directions of ODF, which are aligned with the underlying fiber directions. In each imaging voxel, the directions of fiber tracts are parallel to the directions of maximum diffusion that are defined as local maxima of the ODF [18, 19, 23, 24, 31, 32]. In the next section, the whole process of PSO-Powell hybrid algorithm was described in detail, which was applied to extract the fiber directions through finding the peaks of ODF.

### 2.2. PSO-Powell Hybrid Optimization

Hybrid strategies for optimization are implemented by combining a heuristic algorithm with a mathematical algorithm. This strategy increases reliability in comparison to mathematical methods and increases efficiency in comparison to pure heuristics algorithms [33]. In this work, PSO is coupled with the modified Powell's method in order to obtain a fast and reliable hybrid algorithm. As the heuristic algorithm, PSO is utilized to cover the entire search space, while modified Powell method, as the mathematical algorithm, starting from a point inside this region, quickly reaches local maxima. The combination makes the hybrid algorithm reliable and at the same time maintains properties which lead to rapid convergence. The PSO algorithm is used to cover the entire search space, identifying the region of local maximum, while Powell algorithm quickly reaches the maximum. Another reason for this choice is the fact that this hybrid algorithm does not require the gradients of ODF [34, 35].

In the optimization of ODF, in order to make PSO go through the domains where the local maxima locate, the* c*_2_ parameter in (2) is assigned to zero [34]. Since the two random factors of rand( ) in (2) could increase the stochastic motion of the particles, we removed them from (2) so that the hybrid algorithm could converge to all local maxima as soon as possible. The revised particles' velocity update equation is described by (3).

(2) Vidk+1=ωkVidk+c1rand⁡ Pid−Xidk+c2rand⁡ Pgid−Xidk,

(3) Vidk+1=ωkVidk+c1Pid−Xidk,

where *ω*(*k*) is the inertia weight, *V*_*id*_ stands for the velocity of particles, *c*_1_ and *c*_2_ represent learning factors, rand( ) represents a random value between 0 and 1, *P*_*id*_ is the optimal position of the *k*^th^ iteration, *Pg*_*id*_ is the global optimal position, and *X*_*id*_ stands for the current position.

Because the construction points located on the spherical surface have no structure or order between their relative locations, we interpolated the query points by triangulating the known ones. This step involves traversing of the triangulation data structure to find the triangle that encloses the query point. Once the point is found, the subsequent step is to compute the value of the query point by the nearest-neighbor interpolation method. The detailed procedure of PSO-Powell hybrid searching algorithm is outlined as follows.

**Step 1 .**

Initialize the positions for a swarm of particles of size *N*, and initialize the parameters including the maximum number *t*_max_ of PSO iterations, the maximum number *M* of hybrid iterations, the precision *ε* of Powell searching, and the precision *P* of PSO searching.

**Step 2 .**

Evaluate the fitness of each particle.

**Step 3 .**

If the total number of iterations is greater than *M*, we would stop the iteration and output all the local maxima. Otherwise, turn to Step 4.

**Step 4 .**

If *t* ≤ *t*_max_ (*t* is the number of PSO iterations), the speed and position of the particles would be updated according to (3) and (4). And then *P*_*id*_ and *Pg*_*id*_ should be updated at the same time.

(4) $$\begin{array}{c} X_{id}\left(\;k+1\;\right)=X_{id}\left(\;k\;\right)+V_{id}\left(\;k+1\;\right). \end{array}$$

**Step 5 .**

If Tolerance_PSO < *P*, then we search the extrema for the particles of last PSO iteration using modified Powell method.

(5.1) The particles of the last PSO iteration are considered as the initial points, *X*(0) ∈ *S*. The directions *d*(*i*) (*i* = 0,1,…, *D* − 1) are linearly independent. Generally, *d*(*i*) are set along the directions of axes. Let *k* = 0.
(5.2) Starting from *X*(0), *X*(1), *X*(2),…, *X*(*D*) are obtained by the linear search along the directions of *d*(0), *d*(1),…, *d*(*D* − 1):
  (5) fxi+αidi=minα∈S⁡fxi+αdi,xi+1=xi+αdi,i=0,1,…,D,
where *α*_*i*_ and *α* denote the step length. And *α*_*i*_ is obtained by the linear search.
(5.3) Let *d*^*D*^ = *x*^*D*^ − *x*^0^. If ‖*d*^*D*^‖ ≤ *ε*, *x*^*D*^ is the solution. Otherwise, starting from *x*^*D*^, a new solution *x*^*D*+1^ would be found along *d*^*D*^ by the linear search.
(5.4) The parameter *tl* corresponding to the maximum drop is determined through
  (6) $$\begin{array}{c} f\left(\;x^{tl}\;\right)-f\left(\;x^{tl+1}\;\right)=\underset{0\leq i\leq D}{\max}\left\{\;f\left(\;x^{i}\;\right)-f\left(\;x^{i+1}\;\right)\;\right\}. \end{array}$$
(5.5) If
  (7) fx0−2fxD+f2xD−x0≥2fxtl−fxtl+1

*d*^0^, *d*^1^,…, *d*^*D*−1^ would be still linearly independent, and they are still the search directions of the next iteration. Let *x*^0^ = *x*^*D*+1^, *k* = *k* + 1, then go to (5.2).

(5.6) If (7) is false, *d*^0^, *d*^1^,…, *d*^*D*−1^ are linearly dependent. Let *d*^*tl*+*i*^ = *d*^*tl*+*i*+1^  (*i* = 0,1,…, *D* − *tl* − 1), *x*^0^ = *x*^*D*+1^, and *k* = *k* + 1; then go to Step (5.2).

**Step 6 .**

The new extremum is added to the extreme set.

**Step 7 .**

Reinitialize particle position and velocity. Then go to Step 2.

In this hybrid method, the term of Tolerance_PSO < *P* is the condition of the transformation from PSO to Powell search, in which Tolerance_PSO represents the tolerance of PSO optimization and *P* stands for the Powell searching threshold value. In the construction of ODF, some noise would be introduced. We could select a normalized ODF value as the thresholding, and this could avoid selecting small peaks that may appear due to noise and transformation [27].

This hybrid algorithm could search all the peaks in the feasible region through revised PSO and has the ability of global search for all extremum points. With the strong local search ability of modified Powell method, the accuracy and convergence speed of the hybrid algorithm are improved. The algorithm only uses the value of the ODF without the need to calculate derivatives. In this work, the parameters of the hybrid algorithm are set as follows according to [34, 35]. PSO population size is 100. The max inertia weight is 0.2, and the min inertia weight is 0.1. The acceleration factor *c*_1_ is 0.5. The maximum number of PSO iterations is 120. The searching threshold value *P* is 0.02. The maximum number of PSO-Powell hybrid iterations is 20.

## 3. Results

We utilized multitensor simulated datasets, Tractometer datasets, and in vivo datasets to evaluate the methods for extracting fiber orientations, including ball-stick, modified Powell, diffusion decomposition, and PSO-Powell model. The angular deviations for the four methods were compared and the results proved the validity and feasibility of PSO-Powell hybrid algorithm.

### 3.1. Simulation Study

**Figure 1:**
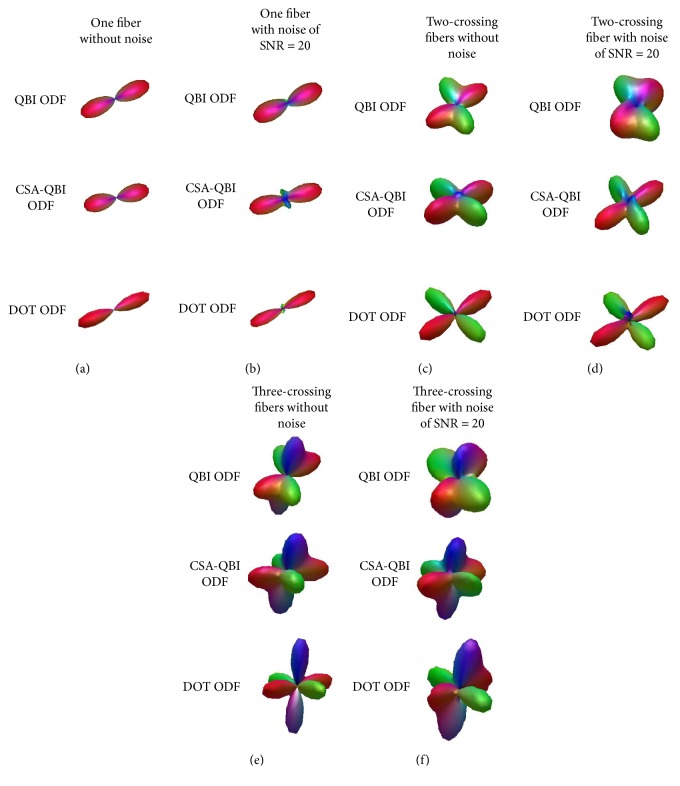
ODFs constructed with QBI, CSA-QBI, and DOT. (a) Single-fiber ODFs without noise. (b) Single-fiber ODFs with noise of SNR = 20. (c) Two-fiber-crossing ODFs without noise. (d) Two-fiber-crossing ODFs with noise of SNR = 20. (e) Three-fiber-crossing ODFs without noise. (f) Three-fiber-crossing ODFs with noise of SNR = 20.

**Figure 2:**
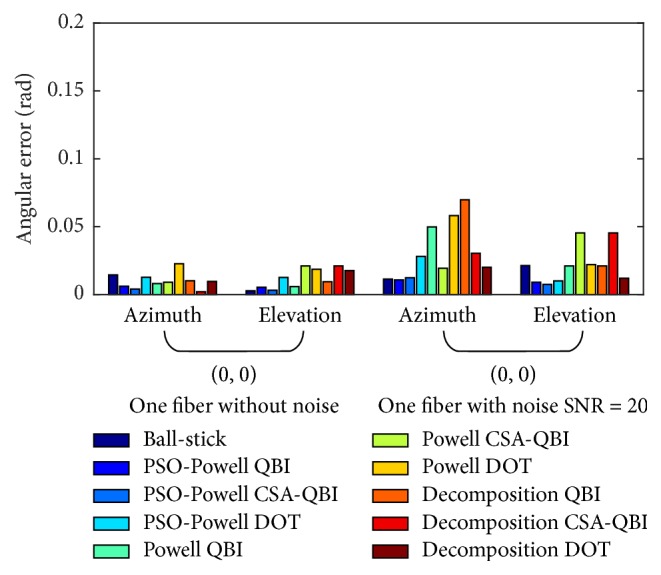
Bar graph illustrating the angular deviations of fiber directions extracted from single-fiber ODFs constructed with QBI, CSA-QBI, and DOT using the methods of PSO-Powell, modified Powell, and decomposition. The ball-stick model directly extracts the directions from DWI raw signals. The azimuth and elevation of the synthetic single fiber are (0, 0).

**Figure 3:**
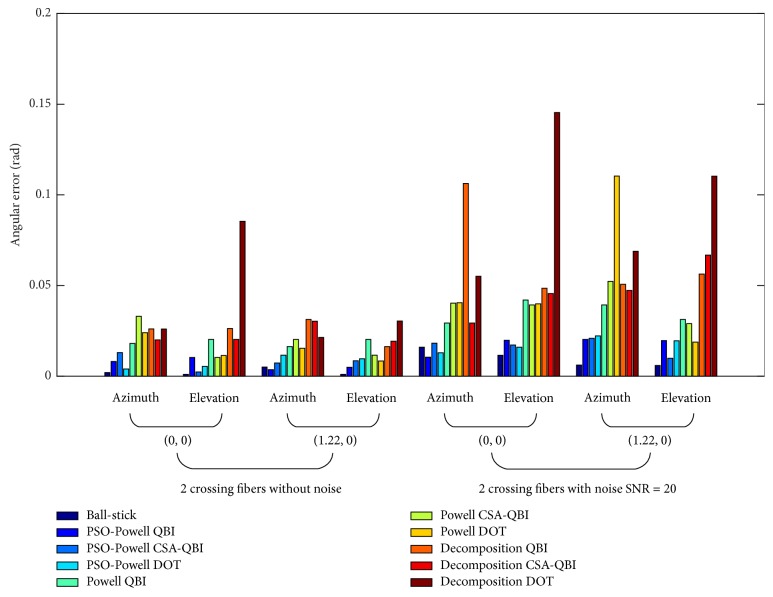
Bar graph illustrating the angular deviations of fiber directions extracted from two-crossing-fiber ODFs constructed with QBI, CSA-QBI, and DOT using the methods of PSO-Powell, modified Powell, and decomposition. The ball-stick model directly extracts the directions from DWI raw signals. The azimuths and elevations of the synthetic two-crossing fibers are (0, 0) and (1.22, 0).

**Figure 4:**
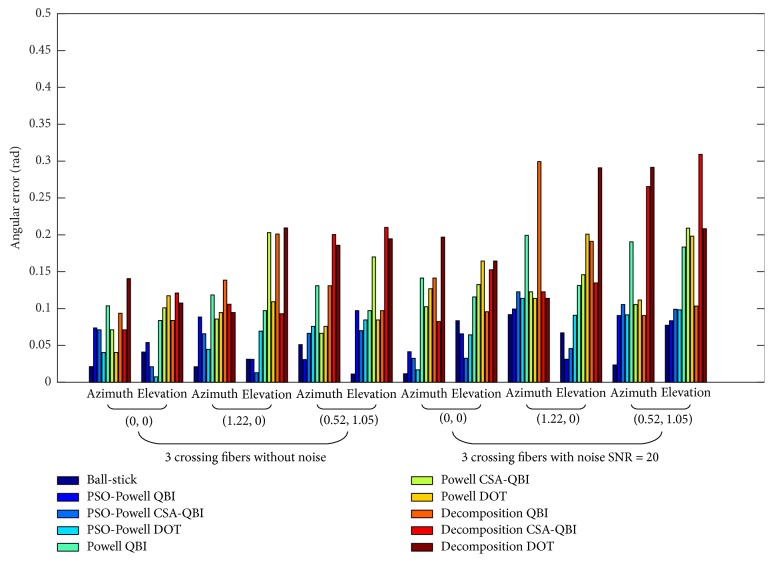
Bar graph illustrating the angular deviations of fiber directions extracted from three-crossing-fiber ODFs constructed with QBI, CSA-QBI, and DOT using the methods of PSO-Powell, modified Powell, and decomposition. The ball-stick model directly extracts the directions from DWI raw signals. The azimuths and elevations of the synthetic three-crossing fibers are (0, 0), (1.22, 0), and (0.52, 1.05).

The synthetic datasets were acquired using the multitensor model [36], which leads to an analytical computation of exact ODF. For a given *b*-value of 3000 s/mm^2^, noise level of SNR = 20 [37], and 64 encoding directions that uniformly are distributed on the unit sphere, we generated DWI raw signals. The simulation parameters of the synthetic datasets about one single-fiber and two and three crossing fibers are shown in Table 1. After the ODF fields were constructed with QBI, CSA-QBI, and DOT, we applied three algorithms including PSO-Powell, modified Powell with sliding windows, and diffusion decomposition to extract the fiber orientations. The constructed ODFs were displayed in Figure 1. The ball-stick model is a simplified model for multiple tensors, and the fiber orientations are directly estimated from DWI raw signals. We directly applied it to the comparison in Figures 2–4.

Figures 2, 3, and 4 show the angular deviations of ball-stick, PSO-Powell, modified Powell, and diffusion decomposition applied to synthetic ODFs without noise and with noise of SNR = 20. The legends located on the right side denote the angular deviations of the evaluated methods. Ball-stick model directly resolves the fiber directions from raw DWI signals. The other three methods were used to extract fiber directions from ODFs constructed with QBI, CSA-QBI, and DOT. The azimuth and elevation deviations were separately grouped for quantitative comparison. As shown in the figures, the diffusion decomposition with QBI ODF has the highest angular deviation for the estimated azimuth *θ* and elevation *φ*. The results of the modified Powell method show better accuracy for noised and noiseless ODFs. Its angular deviation may be due to ODF construction incompatibility and sliding windows radius, and the error can be reduced in low SNR conditions. The ball-stick model performs best for single-fiber and two-crossing-fiber ODFs (Figures 2 and 3) but worse for three-crossing-fiber ODF (Figure 4). In sum, PSO-Powell method showed substantial better performance than other methods. The overall results suggest that PSO-Powell method can be applied to the fiber orientations extraction from ODF fields constructed with QBI, CSA-QBI, and DOT. From the above quantitative comparisons, the hybrid algorithm was shown to be reliable and to perform better than the ball-stick model, modified Powell, and ODF decomposition.

Tables 2–4 show the convergence time of the tested methods for extracting fiber orientations from ODFs constructed with QBI, CSA-QBI, and DOT. From these tables, we can draw the conclusion that PSO-Powell got the better convergence speed than modified Powell and diffusion decomposition. Because ball-stick model resolves the orientations through nonlinear curve fitting in least-squares sense under the condition that the number of diffusion compartments is given, it requires significantly less time. Powell's method takes the longest time because of the repeated application of the sliding window, and the radius is 0.4. In diffusion decomposition, much time must be spent on the iterative regression analysis. These methods were tested on the PC equipped with Intel Core i5-3337U, 4 G RAM, and Windows 10.

### 3.2. Phantom Study

The proposed method was also evaluated on the phantom of Tractometer, which is popular in fiber tracking test. The DWI datasets of Tractometer were acquired on the 3T Tim Trio MRI systems, equipped with a whole body gradient coil, a whole body transmit coil, and a 12-channel head receive coil. A single-shot diffusion-weighted twice refocused spin echo-planar pulse sequence was used to perform the acquisitions in order to compensate for the first order Eddy currents. For each acquisition, there were 64 uniformly distributed diffusion-weighted measurements and one *b* = 0 image, with two repetitions. The spatial resolution for the datasets is 3mm × 3mm × 3mm and 3 slices were acquired. Specific parameters are as follows: field of view 19.2 cm, matrix size 64 × 64, read bandwidth 1775 Hz/pixel, partial Fourier factor of 6/8, GRAPPA factor of 2, TR = 5 s, TE = 102 ms for *b*-value = 2000 s/mm^2^, and corresponding SNR of DWI was estimated to be 1.1. The *b* = 0 image has SNR of approximately 15.8 [38].

**Figure 5:**
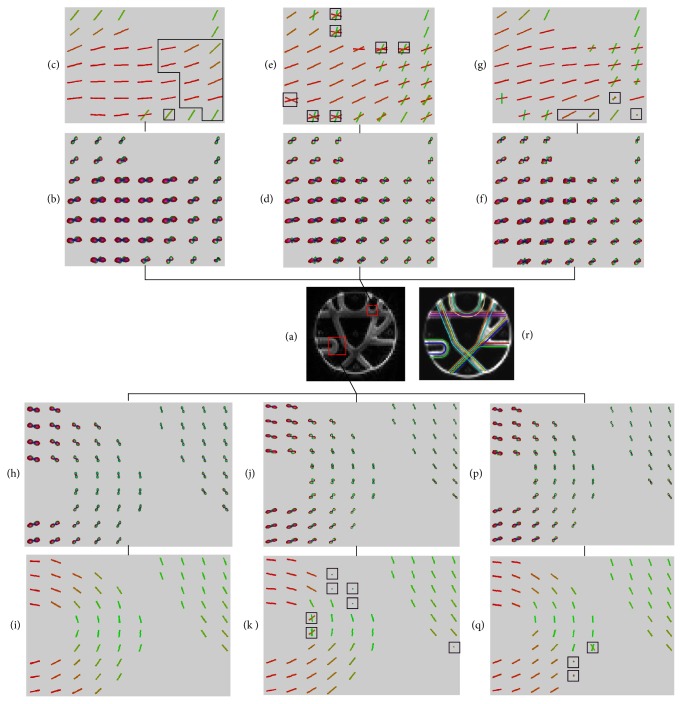
Fiber orientations extracted from ODF fields of Tractometer. In (a), two ROIs are marked out by red squares. (b) and (h) are ODF fields constructed with QBI. (d) and (j) are ODF fields constructed with CSA-QBI. (f) and (p) are ODF fields constructed with DOT. (c), (e), (g), (i), (k), and (q) show the fiber directions extracted from (b), (d), (f), (h), (j), and (p), respectively. (r) shows the ground truth fibers of Tractometer phantom. The directions marked out by black polygons do not correspond with the ground truth.

Figure 5 shows visual comparison of the fiber orientations extracted from ODF fields constructed with QBI, CSA-QBI, and DOT. The ROI regions were marked out by the red squares in Figure 5(a). The top-right ROI contains two-crossing fibers, and the bottom-left ROI contains single fibers. From Figure 5(c), we could see that the marked voxels lost one fiber orientation. A possible cause to this error can be due to the discrepancy between QBI and the actual diffusion pattern in the phantom. In Figure 5(e), the voxels marked by black polygons got some more false directions. In Figure 5(g), the four marked voxels have false orientations, and this is due to the fact that DOT would lead to lager false peaks. In Figure 5(i), the single-fiber directions were correctly estimated. In Figure 5(k), seven voxels have false directions. In Figure 5(q), three voxels have false directions. Overall, in crossing-fiber regions, PSO-Powell method has best performance on the ODF field constructed with DOT. In single-fiber regions, the method stands out on the ODF field constructed with QBI.

### 3.3. In Vivo Study

In order to further illustrate the effectiveness of PSO-Powell hybrid algorithm on neural fiber orientation extraction, we applied the algorithm to in vivo DWI datasets. The whole-brain HARDI was performed on an adult male volunteers (25 years old) using a 3T Signa EXCITE scanner equipped with an eight-channel phased-array head coil. A multislice single-shot echo-planar spin echo pulse sequence was employed to obtain attenuated signals at a diffusion weighting of *b* = 3000 s/mm^2^, where the diffusion-encoding directions were distributed uniformly over the surface of a sphere using electrostatic repulsion. An additional acquisition without diffusion weighting at *b* = 0 s/mm^2^ was also obtained. The total scan time for whole-brain acquisition of 64 diffusion-encoding directions was 19.3 min with TR/TE 18 s/84 ms and isotropic 2 mm voxel resolution (FOV 260 × 260 mm, matrix 140 × 140, 92 interleaved slices with 2mm slice thickness and no gap).

**Figure 6:**
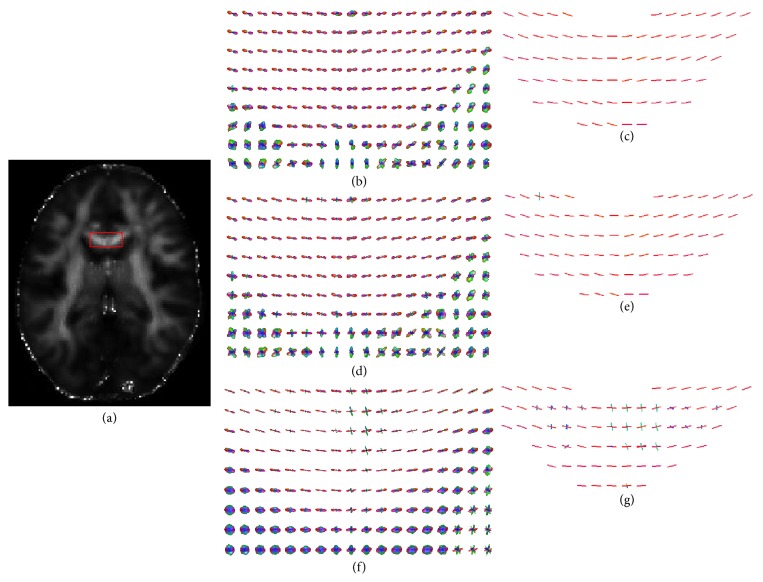
Fiber orientations of CC extracted from ODF fields constructed with QBI, CSA-QBI, and DOT. (a) is the FA map of the 50th slice of in vivo HADRI dataset. (b), (d), and (f) are the ODF fields constructed with QBI, CSA-QBI, and DOT, respectively. (c), (e), and (g) are the vector map extracted from (b), (d), and (f), respectively.

Figure 6 visually shows the fiber orientations estimated from ODF fields constructed with QBI, CSA-QBI, and DOT in the region of the corpus callosum (CC) that is a structure that connects the left and right cerebral hemispheres. After the ODFs were computed, the condition of FA ≥ 0.20 was applied to segment WM tissue out to calculate fiber directions. The results of Figures 6(c) and 6(e) are consistent with known anatomy. In Figure 6(g), some voxels have two-crossing directions, and this is mainly because that DOT has evoked false peaks into ODF due to the impact of MRI Rician noise.

**Figure 7:**
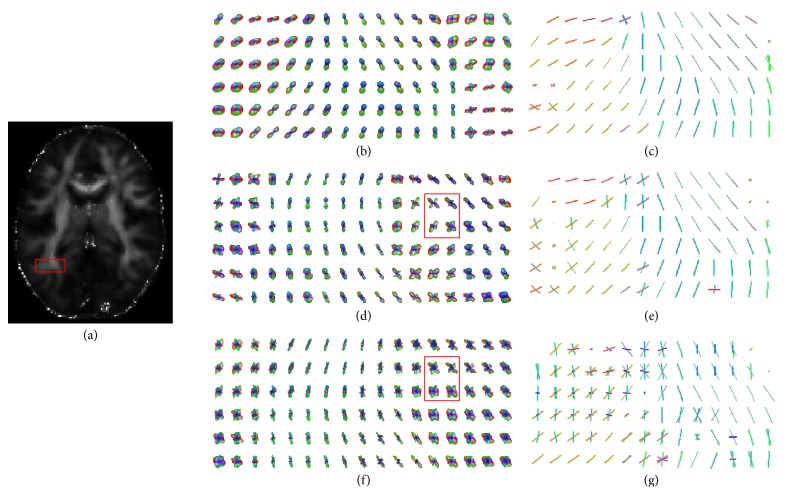
Fiber orientations extracted from ODF fields constructed with QBI, CSA-QBI, and DOT. (a) is the FA map of 50th slice. (b), (d), and (f) are the ODF fields constructed with QBI, CSA-QBI, and DOT, respectively. (c), (e), and (g) are the vector map extracted from (b), (d), and (f), respectively. The ODFs marked out by red rectangles contain spurious peaks coming from computation models.

Figure 7 shows the fiber orientations estimated from ODF fields constructed with QBI, CSA-QBI, and DOT in the ROI region through which cuneus fibers, vertical occipital fasciculi, and superior longitudinal fasciculi pass. This region contains many multimodal ODFs. In Figure 7(g), crossing-fiber directions can all be clearly identified. By contrast, Figures 7(c) and 7(e) only reflect one fiber direction mainly. In the calculation, the directions along which the peak of ODF was less than the mean value were discarded [23], just like the ODFs marked out by red rectangles in Figures 7(d) and 7(f).

## 4. Discussion

The Powell-PSO hybrid algorithm overcomes the shortcomings of PSO algorithm, such as poor accuracy and slow convergence speed. At the same time, it keeps the merits of Powell method, such as computational accuracy and convergence rate. By searching all the peaks in the feasible region, the hybrid algorithm has the ability of searching all the local maximums of ODFs. Since the algorithm only uses the value of the function information without the need to calculate derivatives, it is suitable for solving the problem of differentiable and nondifferentiable multifield optimization (MFO) [35].

The comparisons with ball-stick model, modified Powell, and diffusion decomposition methods show that this hybrid algorithm is effective in searching the diffusion directions from ODFs. But when ODFs have more spurious local maximums due to noise or computational models, more hybrid iterations would be carried out.

In the proposed method, the* c*_2_ parameter is set to zero to increase the stochastic motion of PSO particles. This would make the hybrid algorithm go through the domains where local maxima locate. The other parameters were determined according to [34, 35], such as inertia weight *ω*, acceleration factor *c*_1_, searching threshold *P*, and the number of hybrid and PSO iterations. In simulated experiments, the proposed hybrid method has been tested on single-fiber, two-fiber-crossing, and three-fiber-crossing ODFs. Through quantitative comparisons (Figures 2–4, Tables 2–4), it outperformed other methods. In Tractometer and real experiments, PSO-Powell method has also been verified using single-fiber and crossing-fiber ODFs constructed with QBI, CSA-QBI, and DOT. The results (Figures 5–7) conformed with the ground truth satisfactorily.

The diameter of bundles of axons considered in fiber tractography are on the order of 1 mm, and individual physical fibers on the order of 1–30 *µ*m. At the current resolution of DWI, there are between one-third and two-thirds of imaging voxels in the human brain WM that contain fiber crossing bundles [1, 8]. But higher resolution of fiber directions comes at the cost of higher susceptibility to noise. The low detection rate and accuracy of local maximums of ODFs at critical crossing angles are well-known problems.

Theoretically, there are two types of parameters that can be extracted from ODFs: the orientation of each fiber population and its volume fractions. Having estimated the orientations of the various fiber populations, the corresponding volume fractions could be computed by finding the set of weighting factors providing the best fit of the weighted sum of their respective signal attenuation profiles to the actual measured signal attenuation. Further study should be conducted to propose a more mature model for computing the fiber volume fractions, meanwhile identifying the fiber orientations.

HARDI has become a common tool for the reconstruction of WM architecture by ODF. In recent years, numerous tracking methods based upon HARDI have emerged in order to overcome the shortcomings of ADC and DT which has no the ability to resolve complex WM architecture such as fiber crossing, branching, and kissing. Furthermore, HARDI is more time-saving than DSI because it just needs to sample on a spherical surface in *q*-space. Due to the fact that higher sensitivity to crossing fibers results in higher sensitivity to noise, the false fiber orientations created by noise can cause substantial error in fiber tracking [39]. In order to increase the accuracy of local maximum detection of ODFs and avoid leaving out nondominant diffusion peaks, we should take into account information from neighboring voxels in further research. In order to better estimate the fiber orientations, we may present a straightforward yet effective method for the smoothing, regularization, and sharpening of diffusion profiles in crossing areas of ODF fields obtained from DWI datasets.

## 5. Conclusion

In this work, a novel hybrid method for determining the principal directions of the ODF is proposed by combining the PSO and the modified Powell search. The method tends to extract fiber orientations from ODF fields, leading to no less resolution, but with fewer and smaller spurious peaks, particularly at low SNR. The method can improve the estimation of fiber orientations in heterogeneous WM regions and boost the reliability of fiber tracking. On the basis of the quantitative analyses with the synthetic and phantom datasets, we can conclude that this method is promising to improve the estimation accuracy of fiber orientations from ODF. The experiments with data from a healthy human subject, acquired under clinical imaging conditions, show the method's potential to notably optimize the reconstruction of noncrossing and crossing-fiber orientations. This method may improve the tracking for the construction of brain structural connectivity network.

## Figures and Tables

**Table 1:** Simulation parameters of multitensor model.

	*b*-value (s/mm^2^)	Volume fraction	(azimuth, elevation) (rad)
1 fiber	3000	1	(0,0)
2 crossing fibers	3000	0.5, 0.5	(0,0), (1.22,0)
3 crossing fibers	3000	0.33, 0.33, 0.33	(0,0), (1.22,0), (0.52,1.05)

**Table 2:** Convergence time of extracting fiber orientation from one-fiber ODF.

One-fiber ODF	Ball-stick	Modified Powell QBI/CSA-QBI/DOT	Diffusion decomposition QBI/CSA-QBI/DOT	PSO-Powell QBI/CSA-QBI/DOT
Noise-free	1.12 s	82.15 s/78.03 s/84.09 s	77.63 s/68.27 s/70.05 s	40.17 s/44.79 s/41.45 s
SNR = 20	1.16 s	85.06 s/78.65 s/87.27 s	75.91 s/69.09 s/70.31 s	43.93 s/49.48 s/44.39 s

**Table 3:** Convergence time of extracting fiber orientation from two-fiber ODF.

Two-fiber ODF	Ball-stick	Modified Powell QBI/CSA-QBI/DOT	Diffusion decomposition QBI/CSA-QBI/DOT	PSO-Powell QBI/CSA-QBI/DOT
Noise-free	1.12 s	81.42 s/80.39 s/90.15 s	88.81 s/90.15 s/87.78 s	43.45 s/42.97 s/41.35 s
SNR = 20	1.17 s	87.15 s/81.76 s/92.80 s	93.90 s/93.43 s/90.27 s	45.96 s/44.77 s/45.28 s

**Table 4:** Convergence time of extracting fiber orientation from three-fiber ODF.

Three-fiber ODF	Ball-stick	Modified Powell QBI/CSA-QBI/DOT	Diffusion decomposition QBI/CSA-QBI/DOT	PSO-Powell QBI/CSA-QBI/DOT
Noise-free	1.13 s	82.57 s/82.17 s/87.03 s	102.27 s/98.11 s/90.83 s	46.60 s/45.73 s/50.52 s
SNR = 20	1.21 s	92.80 s/83.74 s/91.70 s	121.95 s/110.25 s/117.12 s	50.16 s/48.96 s/50.62 s
